# The Long-Term Outcomes after Radical Prostatectomy of Patients with Pathologic Gleason 8–10 Disease

**DOI:** 10.1155/2012/428098

**Published:** 2011-09-16

**Authors:** Dan Lewinshtein, Brandon Teng, Ashley Valencia, Robert Gibbons, Christopher R. Porter

**Affiliations:** Section of Urology and Renal Transplantation, Virginia Mason Medical Center, 1100 Ninth Avenue, C7-URO, Seattle, WA 98101, USA

## Abstract

*Background*. We explored the long-term clinical outcomes including metastases-free survival and prostate cancer-specific survival (PCSS) in patients with pathologic Gleason 8–10 disease after radical prostatectomy (RP). *Methods*. We report on 91 patients with PCSS data with a median followup of 8.2 years after RP performed between 1988 and 1997. Cox regression and Kaplan-Meier analysis were used to evaluate year of surgery, pathologic stage, and surgical margin status as predictors of PCSM. *Results*. Median age was 65 years (IQR: 61–9), and median PSA was 9.7 ng/ml (IQR: 6.1–13.4). Of all patients, 62 (68.9%) had stage T3 disease or higher, and 48 (52.7%) had a positive surgical margin. On multivariate analysis, none of the predictors were statistically significant. Of all patients, the predicted 10-year BCR-free survival, mets-free survival, and PCSS were 59% (CI: 53%–65%), 88% (CI: 84%–92%), and 94% (CI: 91%–97%), respectively. *Conclusions*. We have demonstrated that cancer control is durable even 10 years after RP in those with pathologic Gleason 8–10 disease. Although 40% will succumb to BCR, only 6% of patients died of their disease. These results support the use of RP for patients with high-risk localized prostate cancer.

## 1. Introduction

Since the advent of widespread prostate-specific antigen (PSA) testing in the late 1980s and early 1990s, PC detection has increased with a concomitant downward-shift in stage [1, 2]. In addition to PSA, the introduction of the anatomic radical retropubic prostatectomy (RP) [3, 4], breakthroughs in radiotherapy delivery [5], and systemic chemotherapeutic agents [6] have resulted in a 30% mortality reduction [7]. Despite the stage shift and treatment improvements, 15% of contemporary patients will present with high-risk localized prostate cancer [8]. Contemporary high-risk disease is generally defined by high Gleason score rather than elevated PSA or advance stage due PSA testing and digital rectal examination [8, 9]. Unfortunately, 50% of patients with high-risk disease will succumb to biochemical failure within 10 years [10, 11]. 

Although there are no randomized trials that support the use of RP for patients with high-risk disease, recent retrospective studies lend credence to the use of RP as an effective therapy for this group of patients [12, 13]. The renewed interest is based on multiple benefits of surgery. First, it provides excellent local control. Second, it better defines the extent of disease than biopsy alone [14]. Third, with prostate removal, PSA failures can be more easily detected. Fourth, radiation can be given in the adjuvant or salvage setting whereas surgery after radiation is associated with high complication rates [15]. 

In that context, we hypothesized that patients with pathologic Gleason 8–10 disease may have better long-term clinical outcomes after RP than previously thought. Thus we explored the long-term clinical outcomes including metastases-free survival and prostate cancer-specific survival (PCSS) in these high-risk patients after RP.

## 2. Materials and Methods

We retrospectively analyzed charts of patients who underwent radical prostatectomy (RP) between 1988 and 1997 at Virginia Mason Medical Center (VMMC). No patient received neoadjuvant therapy. One of the authors (R. Gibbons) logged clinical and pathological data into a prospective database from 1988 to 1999. After 1999, the records were maintained electronically with institutional review board (IRB) approval. PSA testing began in 1988 at VMMC. All patients were operated between 1988 and 1997. We subselected for a cohort of 91 patients that had pathologic Gleason 8 disease or higher and had postoperative PSA data available. Of all patients, 66 (72.5%) underwent radical retropubic prostatectomy (RRP) and 25 (27.5%) underwent radical perineal prostatectomy (RPP). All specimens were evaluated by VMMC pathologists. Specimens were processed as half mount specimens and serially sectioned at 5 mm intervals. Alternate 5 mm sections were fixed in formalin and were paraffin embedded. From 1988 to 1992 pathological tumor stage was recorded according to the Whitmore-Jewett classification and later converted to the 1992 AJCC staging guidelines [16, 17] From 1992 to 1997 the 1992 AJCC staging system was used for clinical and pathological staging. 

From 1992 to 1997 tumors were routinely classified according to the Gleason grading system [18]. Before 1992 tumor grade was recorded as well differentiated (I), moderately differentiated (II), and poorly differentiated (III). To recode these data we followed the paradigm outlined by Roehl et al., in which well-differentiated tumors are classified as Gleason sum 3, moderately differentiated tumors are assigned Gleason sum 6, and poorly differentiated tumors are assigned Gleason sum 9 [19]. Positive surgical margins were recorded as presence of cancer cells against the inked resection margin. 

Serum PSA testing was initiated at VMMC in 1988. Since that time patients were followed at least quarterly for 2 years, then at least biannually for 2 years, and then at least annually. Biochemical recurrence was defined as PSA greater than 0.1 ng/mL. Metastases were diagnosed based on technetium-99m-based bone scintigraphic studies, and computed tomography cross-sectional imaging was used in equivocal cases. Adjuvant or salvage hormonal and/or radiotherapy were delivered according to individual surgeon preference. Adjuvant therapy was defined as adjunctive radiotherapy in the absence of PSA recurrence (PSA < 0.1 ng/mL) given within 6 months of surgery. Neoadjuvant hormonal therapy was defined as hormone delivery prior to surgery. Cause of death was ascertained according to detailed chart review or was obtained from the VMMC cancer registry. The cancer registry uses links with the Washington State Death Certificate Office. PC must be the first listed cause of death on the certificate for a patient to be classified as having died of PC. 

Prostate cancer-specific mortality was analyzed with univariate and multivariate Cox regression models based on preoperative and operative factors. Predictors included year of surgery, 1992 AJCC pathological stage, and surgical margin status. 

Actuarial analyses addressed the outcomes of PSA recurrence, distant recurrence, PCSS, and overall survival. In analyses of PCSS, patients without evidence of progression were censored at the time of last followup. A biochemical recurrence event for the purposes of Kaplan-Meier analysis was defined as a PSA of 0.1 ng/mL or the delivery of radiotherapy or hormonal therapy after later than 6 months after surgery. Kaplan-All statistical tests and figures were performed with S-PSS (2009) and statistical significance was set at 0.05.

Patients who died of other causes were censored at time of death. PSA recurrence-free data were censored if radiotherapy and/or hormonal therapy were delivered before PSA recurrence.

## 3. Results

Clinical and pathologic characteristics are shown in Table 1. The median followup was 8.2 years (interquartile range [IQR]: 4.5–12.5 years). The median age of the 91 person cohort was 65 years (IQR: 61–69 years) and the median PSA was 9.7 ng/mL (IQR: 6.1–13.4). At time of pathological analysis after RP, 62 (68.9%) had stage T3 disease or higher, 23 (25.3%) had pathologic Gleason 9 or higher, and 48 (52.7%) had a positive surgical margin.

Table 2 shows the univariate and multivariate Cox regression model predicting prostate cancer-specific mortality. On both univariate and multivariate analysis, none of predictors remained statistically significant (*P* > 0.05). 

**Figure 1:**
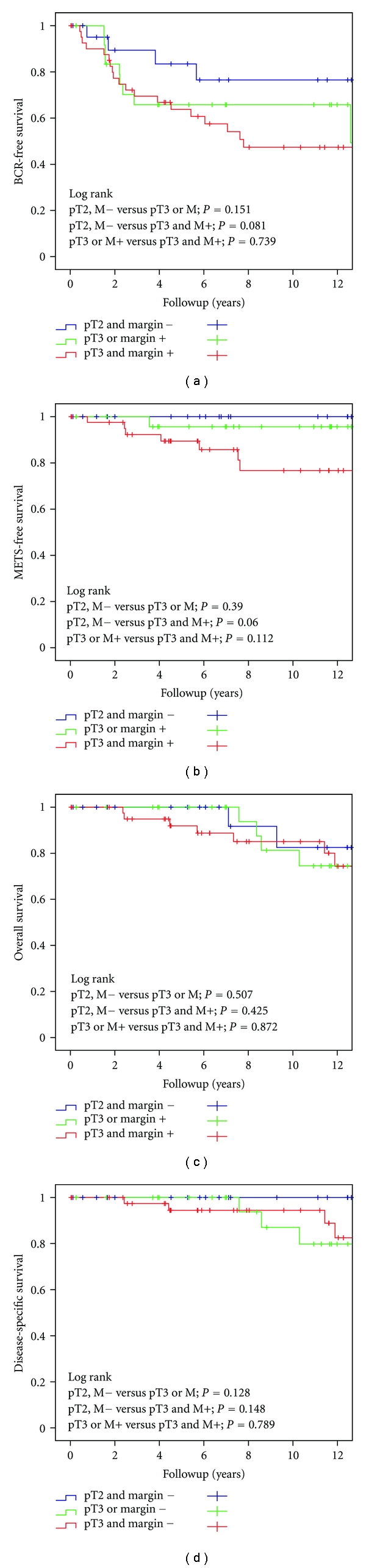
Kaplan-Meier estimates of BCR (a), distant recurrence (b), overall survival (c), and disease-specific survival (d) according to stage and surgical margin status.

Figure 1 graphically displays Kaplan-Meier estimates of BCR (a), metastases (b), overall survival (c), and PCSS (d) stratified by pathologic stage and surgical margin status. There was a trend for mean times to BCR (*P* = 0.081), and metastatic disease (*P* = 0.060), to be different between pT2/margin negative patients and pT3/margin positive patients. 

Table 3 provides Kaplan-Meier actuarial estimates for time to BCR, metastases, overall survival and PCSS. Of all patients, the predicted 10-year BCR-free survival, mets-free survival, and PCSS were 59% (CI: 53%–65%), 88% (CI: 84%–92%), and 94% (CI: 91%–97%), respectively. Specifically, the predicted 10-year BCR-free rate was significantly better in those with organ-confined margin negative disease (pT2) than in those with locally advanced (pT3) margin positive disease (77% (CI: 66%–88%) versus 47%(CI: 38%–56%)). The predicted 15-year PCSS was significantly better in those with organ-confined margin negative disease (pT2) than in those with locally advanced (pT3) margin positive disease (100% (CI: 100%-100%) versus 73%(CI: 62%–84%)).

## 4. Discussion

We have demonstrated in a cohort with a median followup time of 8.2 years that cancer control is durable even 10 years after RP in those with pathologic high-grade disease. Although, 41% of patients developed BCR by 10 years, only 12% of patients in this extremely high-risk group progressed to distant metastases, and just 6% of patients actually died of their disease (Table 3). 

When put into context, 59% of all patients with pathologic Gleason 8 disease or higher and 47% of patients with pathologic Gleason 8 disease or higher, pT3 stage and margin positive disease were cured of their disease (no BCR within 10 yr) with primary RP (Table 3). However, cure was not achieved solely with surgery. Of all patients, 11 received solely postoperative radiotherapy, 10 received long-term hormonal therapy, and 9 received both postoperative radiotherapy and long-term hormonal therapy. Clearly, this is a group of patients that will require multimodal therapy to achieve robust durable outcomes.

Our 10-year actuarial disease-specific mortality estimate (6%) was similar to other long-term RP series, including the UCLA group (8%) [20] and Hull et al. (2.4%) [21], and compares favorably with reports that have examined locally advanced disease specifically [12, 22].

It is intriguing that the classical predictors of outcome such as pathological stage and surgical margin status did not reach statistical significance in our multivariate analysis. However, the multivariate effect of these variables on PCSS was not assessed in most other long-term outcome series except that of Stephenson et al. [23]. However, stage was a significant predictor on multivariate analyses of BCR after RP in other series with long-term followup [19, 21] as was surgical margin status [21]. Thus, the positive predictor status of these classic variables with regard to BCR, but their inability to predict long-term PCSM suggest that they may not be important with regard to long-term oncologic control. This assumption will require further study in other long-term followup RP series.

The results of our study must be interpreted within the strengths and limitations of our study. First, our data derive from a single center over 20 years, and involve multiple surgeons. Thus, patient selection and surgical technique certainly differed and possibly may have introduced variation in outcome [24]. Moreover, patients underwent two surgical techniques, namely, RPP and RRP, which may have affected outcome. However, the literature suggests that there is no difference between RPP and RRP oncologic outcomes [25]. Second, we recognize that there is no centralized pathologic review and that therefore contemporary Gleason scores may well be ascribed a higher value [14]. However, all pathology was read at a single tertiary referral center with a high level of GU pathology expertise. Moreover, we relied upon the original pathology report from VMMC to establish the histological differentiation. Gleason scores were not routinely recorded before 1992, and therefore, we relied upon the paradigm used by Roehl to assign Gleason scores to patients undergoing surgery before 1992 [19]. Finally, postoperative use of radiation and androgen deprivation therapy were given at the discretion of the treating physician and may have introduced substantial bias into the interpretation of the results.

## 5. Conclusions

In summary, we have demonstrated in a cohort with a median followup time of 8.2 years that cancer control is durable even 10 years after RP in those with pathologic Gleason 8–10 disease. Although, 41% of patients developed BCR by 10 years, only 12% of patients in this high-risk group progressed to metastases, and just 10% of patients died of their disease. Taken together, these long-term oncologic results support the use of RP for patients with high-risk localized prostate cancer.

## Figures and Tables

**Table 1:** Demographics and pathologic and clinical outcomes.

Characteristic	No. (IQR)
Median followup in years	8.2 (4.5–12.5)
Median age in years	65 (61–69)
Median pre-op PSA	9.7 (6.1–13.4)
Pathologic tumor volume	5.3 (3.0–12.0)
Pathologic Gleason sum	No. (%)
Gleason 8	68 (74.7)
Gleason 9	22 (24.2)
Gleason 10	1 (1.1)
Pathologic stage	
pT2	28 (31.1)
pT3/4	62 (68.9)
Positive margins	48 (52.7)
Lymph node dissection and node status	
Nx	34 (37.4)
N0	49 (53.8)
N1	6 (6.6)
N2	2 (2.2)
Adjuvant radiation	10 (11.0)
Salvage radiation	10 (11.0)
Neoadjuvant ADT	9 (9.9)
Salvage ADT	19 (20.9)
Biochemical recurrence	33 (36.3)
Metastatic disease	8(8.8)
PCSM	9 (9.9)
Total no. patients	91 (100)

ADT: androgen deprivation therapy.

**Table 2:** Binary logistic regression for prostate cancer-specific mortality.

Characteristics	Univariate hazard ratio (95% CI)	(*P* value)	Multivariate hazard ratio (95% CI)	(*P* value)
Pre-op PSA	1.038 (0.970–1.112)	0.278	1.042 (0.953–1.139)	0.366
Gleason (8 versus 9–10)	0.830 (0.160–4.312)	0.825	1.087 (0.106–11.146)	0.944
Stage (pT3/4 versus pT2)	4.000 (0.476–33.645)	0.202	0.627 (0.032–12.429)	0.759
Margin status	1.905 (0.446–8.136)	0.384	4.943 (0.342–71.440)	0.241
XRT received	1.912 (0.433–8.442)	0.392	3.529 (0.356–34.987)	0.281
ADT received	3.207 (0.790–13.007)	0.103	0.703 (0.050–9.948)	0.795
Node status	1.257 (0.127–12.419)	0.845	2.127 (0.059–77.249)	0.680

XRT = adjuvant or salvage radiotherapy; ADT: hormone use for relapse.

**Table 3:** Kaplan-Meier actuarial estimates of BCR-free survival, metastases-free survival, overall survival, and prostate cancer specific survival (PCSS).

Cohort	Overall cohort	pT2/margin −	pT3 or margin +	pT3 & margin +
No at risk/5-year BCR-free survival (CI)	90/0.69 (0.064–0.74)	22/0.84 (0.76–0.90)	25/0.65 (0.55–0.75)	42/0.64 (0.56–0.68)
No at risk/5-year mets-free survival (CI)	90/0.94 (0.91–0.97)	22/1.00 (1.00–1.00)	25/0.96 (0.92–1.00)	42/0.89 (0.84–0.94)
No at risk/5-year overall survival (CI)	90/0.96 (0.94–0.98)	22/1.00 (1.00–1.00)	25/1.00 (1.00–1.00)	42/0.92 (0.88–0.96)
No at risk/5-year PCSS (CI)	90/0.97 (0.95–0.99)	22/1.00 (1.00-1.00)	25/1.00 (1.00-1.00)	42/0.95 (0.91–0.99)
No at risk/10-year BCR-free survival (CI)	47/0.59 (0.53–0.65)	13/0.77 (0.66–0.88)	13/0.65 (0.55–0.75)	21/0.47 (0.38–0.56)
No at risk/10-year mets-free survival (CI)	61/0.88 (0.84–0.92)	16/1.00 (1.00-1.00)	19/0.96 (0.92–1.00)	26/0.77 (0.69–0.85)
No at risk/10-year overall survival (CI)	65/0.84 (0.79–0.89)	16/0.85 (0.75–0.95)	20/0.83 (0.74–0.92)	29/0.85 (0.79–0.91)
No at risk/10-year PCSS (CI)	65/0.94 (0.91–0.97)	16/1.00 (1.00-1.00)	20/0.88 (0.80–0.96)	29/0.95 (0.91–0.99)
No at risk/15-year BCR-free survival (CI)	29/0.52 (0.45–0.59)	8/0.77 (0.66–0.88)	9/0.45 (0.31–0.59)	12/0.47 (0.38–0.56)
No at risk/15-year mets-free survival (CI)	37/0.88 (0.84–0.92)	9/1.00 (1.00-1.00)	12/0.96 (0.92–1.00)	16/0.77 (0.69–0.85)
No at risk/15-year overall survival (CI)	40/0.69 (0.62–0.76)	9/0.85 (0.75–0.95)	12/0.74 (0.62–0.86)	19/0.59 (0.48–0.70)
No at risk/15-year PCSS (CI)	40/0.80 (0.73–0.87)	9/1.00 (1.00-1.00)	12/0.78 (0.66–0.90)	19/0.73 (0.62–0.84)
